# Inter- and Intra-Observer Variability and the Effect of Experience in Cine-MRI for Adhesion Detection

**DOI:** 10.3390/jimaging9030055

**Published:** 2023-02-23

**Authors:** Bram de Wilde, Frank Joosten, Wulphert Venderink, Mirjam E. J. Davidse, Juliëtte Geurts, Hanneke Kruijt, Afke Vermeulen, Bibi Martens, Maxime V. P. Schyns, Josephine C. B. M. Huige, Myrte C. de Boer, Bart A. R. Tonino, Herman J. A. Zandvoort, Kirsti Lammert, Helka Parviainen, Aino-Maija Vuorinen, Suvi Syväranta, Ruben R. M. Vogels, Wiesje Prins, Andrea Coppola, Nancy Bossa, Richard P. G. ten Broek, Henkjan Huisman

**Affiliations:** 1 Department of Radiology and Nuclear Medicine, Radboud University Medical Center, 6525 GA Nijmegen, The Netherlands; 2 Department of Radiology, Rijnstate Hospital, 6883 AD Arnhem, The Netherlands; 3 Faculty of Science and Technology, University of Twente, 7522 NB Enschede, The Netherlands; 4 Department of Radiology and Nuclear Medicine, Maastricht University Medical Center, 6229 HX Maastricht, The Netherlands; 5 Radiology, HUS Diagnostic Center, Helsinki University Hospital, 00014 Helsinki, Finland; 6 Department of Radiology, Medical Spectrum Twente, 7512 KZ Enschede, The Netherlands; 7 Department of Diagnostic and Interventional Radiology, ASST dei Sette Laghi, 21100 Varese, Italy; 8 Department of Radiology, Centro Radiologico Dr Gomez Pereda, 50004 Zaragoza, Spain; 9 Department of Surgery, Radboud University Medical Center, 6525 GA Nijmegen, The Netherlands

**Keywords:** (MeSH), tissue adhesions, magnetic resonance imaging, observer variation

## Abstract

Cine-MRI for adhesion detection is a promising novel modality that can help the large group of patients developing pain after abdominal surgery. Few studies into its diagnostic accuracy are available, and none address observer variability. This retrospective study explores the inter- and intra-observer variability, diagnostic accuracy, and the effect of experience. A total of 15 observers with a variety of experience reviewed 61 sagittal cine-MRI slices, placing box annotations with a confidence score at locations suspect for adhesions. Five observers reviewed the slices again one year later. Inter- and intra-observer variability are quantified using Fleiss’ (inter) and Cohen’s (intra) κ and percentage agreement. Diagnostic accuracy is quantified with receiver operating characteristic (ROC) analysis based on a consensus standard. Inter-observer Fleiss’ κ values range from 0.04 to 0.34, showing poor to fair agreement. High general and cine-MRI experience led to significantly (*p* < 0.001) better agreement among observers. The intra-observer results show Cohen’s κ values between 0.37 and 0.53 for all observers, except one with a low κ of −0.11. Group AUC scores lie between 0.66 and 0.72, with individual observers reaching 0.78. This study confirms that cine-MRI can diagnose adhesions, with respect to a radiologist consensus panel and shows that experience improves reading cine-MRI. Observers without specific experience adapt to this modality quickly after a short online tutorial. Observer agreement is fair at best and area under the receiver operating characteristic curve (AUC) scores leave room for improvement. Consistently interpreting this novel modality needs further research, for instance, by developing reporting guidelines or artificial intelligence-based methods.

## 1. Introduction

Postoperative chronic pain develops in 10–20% of people undergoing abdominal surgery [1,2]. In about half of these patients the pain may be attributable to adhesions [3,4]. Currently, diagnosis and management of chronic postoperative pain related to adhesions is a clinical challenge. Diagnosis of adhesions is commonly established by diagnostic laparoscopy, an invasive procedure with up to 20–30% negative findings [5]. Moreover, in case of severe adhesions, surgical release of adhesions might be complicated by injury to bowels or other organs [6]. Cine-MRI can accurately diagnose and map adhesions, avoiding negative findings and complications during surgery [7,8]. Using such non-invasive techniques to diagnose and map adhesions holds great promise for supporting clinical decision-making and patient selection. In a recent clinical study, adding cine-MRI in the work-up of patients with chronic pain reduced the rate of negative laparotomies to 5% and risk of severe injuries to 2%. Long-term pain results were also excellent with approximately 80% improvement in patients who received surgery [9]. A limitation of cine-MRI is the radiological expertise required for the interpretation of movement patterns in the abdomen. Technically, implementation of cine-MRI seems feasible without any major infrastructure investments. The images can be obtained on a standard 1.5 Tesla scanner and all major manufacturers support the cine sequences. However, application of cine sequences is relatively new in the field of abdominal MRI, and the full potential of functional abdominal cine-MRI is yet to be explored [10,11,12]. At present, it remains unclear what kind of radiological experience is required to accurately detect adhesions on cine-MRI. Few radiologists have extensive experience specifically with abdominal cine-MRI. Potentially, extensive general experience or experience with cine-MRI in other fields (e.g., cardiac MRI) are also helpful in detecting adhesions.

The goal of this retrospective study is to assess the variability and performance of observers with a range of radiological experience in diagnosing adhesions in abdominal cine-MRI. We hypothesize that observers without specific experience are less accurate and show more variability than observers with specific abdominal cine-MRI experience. Additionally, we expect that observers with some experience with cine-MRI perform relatively better than observers to whom cine-MRI is novel.

## 2. Materials and Methods

The dataset comprised a random sample of cine-MRI studies. Studies were retrospectively selected from a consecutive cohort of patients that received a cine-MRI study in 2019 in our expertise center for chronic postoperative pain, which is a collaboration between the Radboud University Medical Center and the Rijnstate Hospital in The Netherlands. Written informed consent was waived by the Institutional Review Board (METC Oost-Nederland, registration number 19082). 

The studies follow the protocol earlier described by Lienemann et al. [13], with some minor improvements due to new scanner hardware. Each cine-MRI sequence uses true-FISP scan, with echo and relaxation times of 1.53 and 3.66 ms, a flip angle of 60°, a matrix size of 192 × 256, slice thickness of 5 mm. No contrast or specific preparation other than movement instructions were applied. A single study consists of 5–7 sagittal and transverse 2D cine slices, covering the entire abdominal cavity. Each cine slice consists of 30 frames with a frame rate of 2.3 frames/s. All studies were obtained with a single 1.5-Tesla scanner (Siemens). Patients were selected for a cine-MRI study based on clinical history and symptoms that led to suspicion of adhesions as the cause for chronic abdominal pain. These criteria resulted in a consecutive cohort of 64 patients who received a cine-MRI study in the year 2019. Based on the accompanying radiology reports, there was no evidence for presence of adhesions in 15 of the 64 patients in the cohort (23%). 

**Figure 1:**
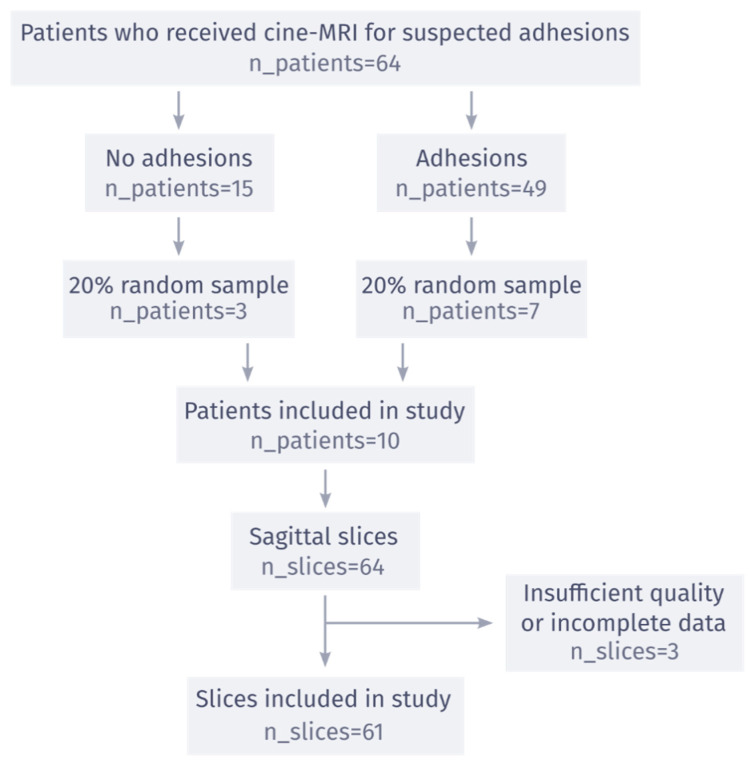
Flowchart of the inclusion process for the observer dataset. A total of 20% of eligible patients was sampled randomly, after splitting them into groups with and without adhesions. All sagittal slices of these patients were included in the study, after exclusion due to insufficient quality or incomplete data from observers.

To remain within the limits of a reasonable time investment from our observers, the final observer dataset was limited to a subset of the consecutive cohort of 64 patients. These 64 patients were randomly sampled from our existing database. Patients were first split into negative (15 patients) and positive (49 patients) groups, based on the overall results of cine-MRI as described in the radiological report. Then, a 20% random sample was taken from each group to preserve the relative prevalence of adhesions of the study population, resulting in a final patient count of 10. In this study, only sagittal slices were included since they contain the most salient information for diagnosis. After exclusion of 2 slices due to insufficient quality and 1 due to incomplete data, the final observer dataset consisted of 61 sagittal cine slices (see Figure 1 for a flowchart). The number of patients in the dataset was chosen such that it would take an inexperienced observer about 4 h to complete the study. In this way it was possible to include a larger number of observers, without requiring significant experience in reading abdominal cine-MRI. 

The study was performed online with an international group of radiologists of varying expertise. All sagittal cine-MRI slices were presented sequentially to the observers, with a corresponding patient identifier, so that observers could tell which slices were from which patient. They viewed the studies in the interactive online observer study platform on the AI development platform https://grand-challenge.org (accessed on date 26 August 2021, see Supplemental Figure S1). For each adhesion they detected, they placed a 2D bounding box annotation and indicated their confidence on a 5-point scale. As training, all observers without experience in adhesion detection received four annotated cases with explanation by one of the consensus readers. This tutorial is openly available online (see Supplemental Figure S1). Observers were blinded for any clinical information and the reference standard. They were informed that there were negative patients in the dataset. 

The reference standard was obtained through a consensus meeting between two experienced observers and a clinical expert. They defined the reference standard, because they are part of the core clinical team of our expertise center and are the main clinical readers and users of cine-MRI in clinical practice. Each of these observers first performed the study by themselves, blinded for the other reviewers’ outcome and clinical data. Afterward, they reviewed each sagittal slice together in a consensus meeting to set the reference standard. During the meeting, cine-MRI slices were reviewed along with the annotations of these three reviewers, using the same interactive online viewer. Discrepancies were resolved by discussion. This meeting resulted in a unanimous patient- and slice-level consensus.

Each observer was placed into three groups, based on their experience. Observers with less/more than 5 years of general experience (including residency) were placed in the low-year/high-year group. Observers who have reviewed less/more than 30 cine-MRI studies of any type were placed in the low-cine/high-cine group. Observers who have reviewed less/more than 1 cine-MRI studies for adhesion detection were placed in the low-adhesion/high-adhesion group. The consensus readers were placed in a separate consensus group. All analyses were performed on a slice level since positive patients did not necessarily have an adhesion visible on every slice. Performance of individual observers compared to the reference standard set by the consensus group was quantified using receiver operating characteristic (ROC) analysis, specifically with the area under the ROC curve (AUC). Observers in the consensus group were excluded from ROC analysis because they set the reference standard on which the ROC curves are based. The ROC curves were constructed by comparing the confidence score given by the observer for each slice to the reference standard for each slice. The number of points in each observer’s ROC curve depended on how many unique confidence levels that observer used when scoring the cases. Additionally, inter-observer variability was quantified with Fleiss’ κ and percentage agreement for each group, including the consensus group. Percentage agreement was calculated by comparing agreement between observers in a single group, without considering the reference standard. When all observers in a group unanimously agreed on a slice, this was counted as agreement. Conversely, all other slices were counted as disagreement. Percentage agreement was the percentage of slices where all observers in a group gave the same diagnosis. Average AUC with 95% confidence interval was calculated for each group, using the iMRMC (Multi-Reader, Multi-Case) software package (v 4.0.3 https://github.com/DIDSR/iMRMC (accessed on 19 February 2023)) [14]. This software package enables statistical ROC comparison between two groups of multiple observers. The z-test included in iMRMC was used to obtain *p*-values for the difference in AUC between the low/high experience groups. For the subset of observers that completed the study twice, Cohen’s κ and percentage agreement were used to quantify intra-observer variability. For both Cohen’s and Fleiss’ κ, values lower than 0 were interpreted as poor agreement, and values higher than 0, 0.2 and 0.4 were interpreted as slight, fair, and moderate, respectively [15]. To binarize confidence scores for the agreement metrics, observers were asked which confidence threshold (on the 5-point scale) they would use in clinical practice to distinguish positive from negative patients. Differences were interpreted as significant when *p* < 0.05. 95% confidence intervals and *p* values for Fleiss’ κ, Cohen’s κ and percentage agreement values were estimated with bootstrapping and permutation tests, respectively. Bootstrapping and permutation tests are solid statistical approaches when the distributions under test are unknown. In our case, we cannot make any parametric assumptions about the distribution of agreement metrics, such as Cohen’s κ. If the distribution of the agreement metrics is known beforehand, parametric tests may provide more statistical power. All statistical analysis, apart from the AUC tests in iMRMC, were performed with Python, version 3.9.10.

## 3. Results

**Figure 2:**
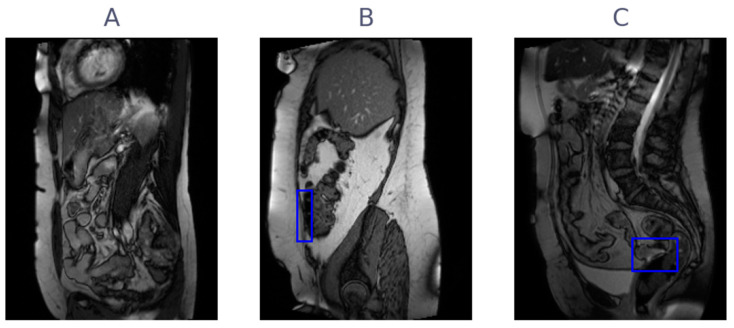
Three examples of cine-MRI slice with typical adhesions, with consensus annotations overlayed as blue boxes. (**A**) A slice without adhesions present, (**B**) a slice with an adhesion to the anterior abdominal wall, and (**C**) a slice with an adhesion in the pelvic area.

The flow of patients in the study is shown in Figure 1. Of the 10 patients included in the observer study, 9 were female. The age of the patients ranged from 30 to 74, with a mean age of 54. Of the seven patients with adhesions indicated in the original radiology report, six patients had adhesions in the lesser pelvis, 2 patients had adhesions between bowel loops, and none had adhesions to the anterior abdominal wall. All scans were acquired in 2019 and were reviewed by 15 observers between March 2020 and October 2020, with 3 observers forming the consensus group. The consensus meeting resulted in a unanimous reference standard, where 7 of 10 patients had adhesions (3 negative), and 19 of 61 slices had adhesions (42 negative). Figure 2 shows three examples of cine-MRI slices, with the consensus annotations shown as blue boxes. Since cine-MRI is a dynamic modality, these examples are best viewed as movies. They are included in the Supplementary Files (Videos S1–S3). A subset of five observers, containing two observers from the consensus group, reviewed the scans a second time between May 2021 and June 2021. The years of general experience, including residency, ranged from 0 to 30, with a mean of 7. The amount of cine-MRI cases reviewed ranged from 0 to 300, with a mean of 52. The amount of adhesion cine-MRI cases reviewed ranged from 0 to 300, with a mean of 32. A detailed overview of the observer experience, group assignment, and clinical significance thresholds is provided in Table 1.

In general, the consensus group shows significantly better agreement compared to the other groups. General and cine experience both improve agreement significantly. The inter-observer Fleiss’ κ and percentage agreement values are shown in Table 2. Specifically, the consensus group had a significantly higher agreement than all other groups (low-year *p* < 0.001; high-year *p* < 0.001; low-cine *p* < 0.001; high-cine *p* = 0.002; low-adhesion *p* < 0.001; high-adhesion *p* < 0.001) and significantly higher Fleiss’ κ than the low-year, low-cine and high-adhesion groups (*p* = 0.008; *p* = 0.005; *p* = 0.03). The high-year group has a significantly higher % agreement and Fleiss’ κ than the low-year group (*p* < 0.001; *p* < 0.001). The high-cine group has a significantly higher % agreement and Fleiss’ κ than the low-cine group (*p* < 0.001; *p* < 0.001). There were no significant differences in % agreement or Fleiss’ κ between the high-adhesion and low-adhesion groups. The intra-observer Cohen’s κ and percentage agreement values are shown in Table 3, showing no clear difference between readers in the consensus group and others. 

**Figure 3:**
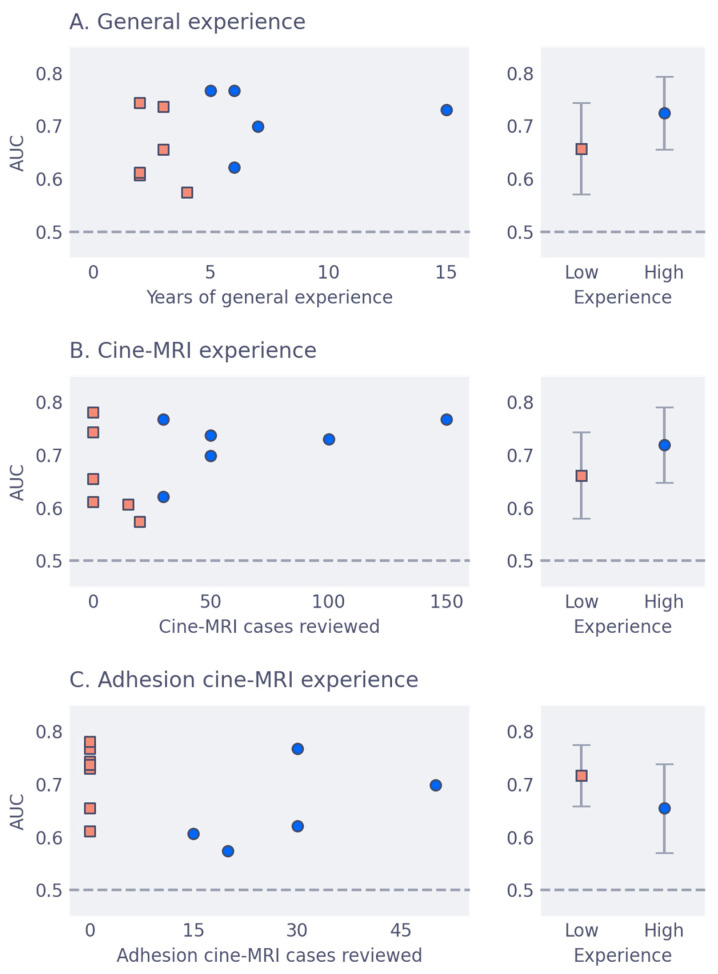
AUC versus: (**A**) Years of general experience, including residency; (**B**) amount of cine-MRI cases reviewed; and (**C**) amount of adhesion cine-MRI cases reviewed. The left column shows individual AUC scores per observer, with with orange squares indicating low experience grouping and blue circles indicating high experience grouping. The right column shows the diagonally averaged AUC for the experience groupings, with bars indicating 95% confidence intervals. This figure excludes the consensus observers since they set the reference standard.

**Figure 4:**
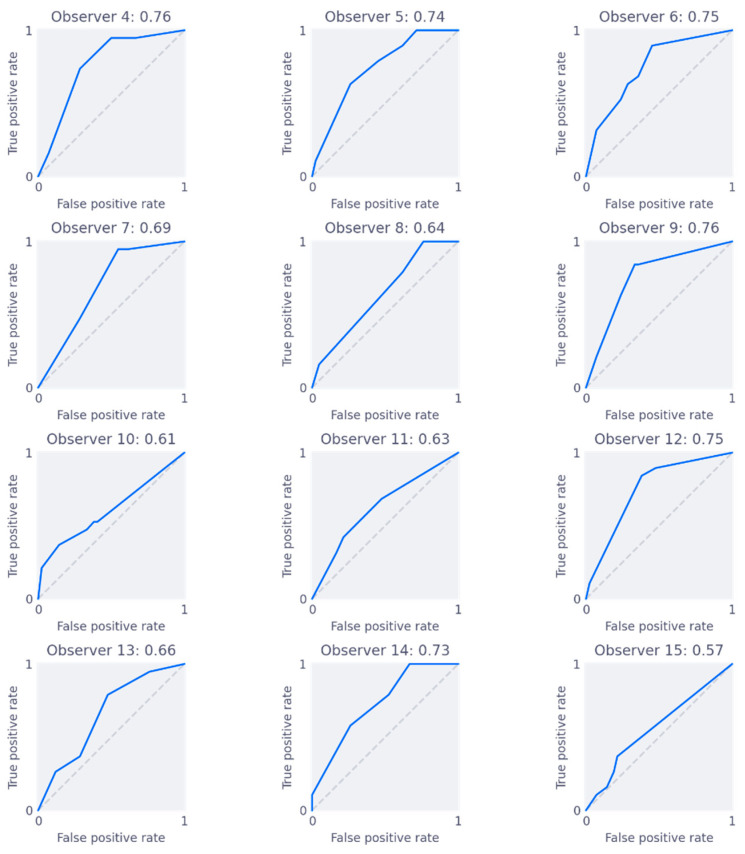
Individual ROC curves for each observer. The title of each subplot shows the observer number, followed by their AUC score. This figure excludes the consensus observers since they set the reference standard.

**Figure 5:**
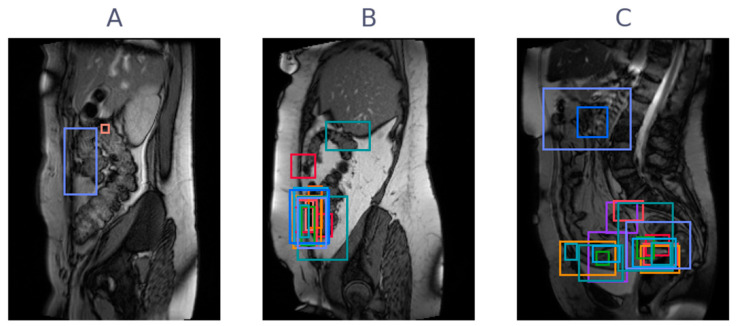
Three examples of cine-MRI slice with varying levels of agreement between observers. Annotations are overlayed as boxes, with a unique color per observer. (**A**) A slice with high agreement for being negative (only two observers annotated), (**B**) a slice with high agreement for being positive in the same location, and (**C**) a slice with many positive annotations but in varying locations.

The area under the receiver-operator characteristic curve (AUC) on slice level versus experience is shown in Figure 3, for the three types of experience considered. The mean AUC for all groups is around 0.7, with general and cine experience trending toward better accuracy. Mean AUC with confidence intervals per group are shown in Table 2. From iMRMC analysis, there were no significant differences in AUC between any of the groups. The individual ROC curves for each reader, along with their AUC scores, are shown in Figure 4. Finally, Figure 5 shows three examples of slices with varying levels of agreement among observers.

## 4. Discussion

This study shows that cine-MRI is a promising modality, with observers without specific experience reaching diagnostic accuracies of 0.75, after a short online tutorial (see Supplemental Figure S1). More general experience or more cine-MRI experience yields significantly better agreement and correlates with higher overall performance, although AUC differences are not significant. Adhesion-specific cine-MRI experience does not result in a significant improvement in agreement or performance in this study. The Fleiss’ κ scores indicate poor to fair agreement across groups and in general are highest for the groupings with higher experience [15]. This indicates that, although experience improves agreement, even in the consensus group there is room for improvement. From the Cohen’s κ values, most observers show moderate agreement with values near 0.5. Observer 5 has a substantially lower κ of −0.11, which may be explained by a learning effect. Observer 5 had very little experience with cine-MRI (only a couple of cases reported) during the first reading, but substantially more (about 20 cases reported) one year later. Considering the ROC analysis, results are acceptable, with improvement possible in all groups: group AUC scores are around 0.7, with individuals scoring near 0.8. In the clinical problem of abdominal pain from adhesions, treatment (surgery) comes with substantial risk. It may therefore be preferred to choose a high-sensitivity operating point on the ROC curve (high confidence threshold), such that false positives are minimized. Overall, the results indicate that general or cine experience helps in achieving good performance, and that on all experience levels, there is room for improvement in terms of consistent rating of abdominal cine-MRI. 

### 4.1. Comparison with the Literature

There is existing literature where diagnostic performance in adhesion detection is studied. This study is the first to address observer variability in abdominal cine-MRI. Existing studies into diagnostic performance in adhesion detection did not perform ROC analysis, but by choosing operating points on ROC curves, an approximate comparison is possible. As example, the average ROC curve of the high-year group is used for selecting operating points (see Supplemental Figure S2 for a visual comparison). Lang et al. studied diagnostic performance with a cohort of 89 patients, with a surgical reference standard [7]. They report a sensitivity/specificity of 93%/25%. The corresponding operating point of the high-year group is 96%/27%. Another study by Zinther et al. includes 60 patients, who all underwent laparoscopic inspection as a gold standard [16]. They report a sensitivity/specificity of 21%/87%, with corresponding operating point 24%/91%. The study of Langbach et al. shows the most balanced results with a sensitivity/specificity of 70%/75%, but only evaluated detecting adhesions to the anterior abdominal wall in a specific patient group [8]. The corresponding operating point of the high-year group is 66%/67%. This shows that the results in this study align with the previous literature and that differences in sensitivity and specificity may be attributed to observers in the literature reading cine-MRI at different operating points. 

### 4.2. Limitations

All scans used in this study were acquired at a single center, with a single scanning protocol. This may reduce the generalizability of the results of this study to other centers or scanning protocols. However, an important benefit of using cine-MRI scans from a single center is that few centers routinely make cine-MRI for adhesions. Thus, including images from our expertise center ensures good scanning quality on which to base radiological judgment.

The study was performed using an online workstation that is considerably simpler than workstations that observers routinely use. It only showed a single sagittal slice at a time, whereas other workstations allow simultaneous viewing of all sagittal cine-MRI slices, together with a coronal overview. Several observers reported that this was inconvenient and that it may have affected their results slightly.

The reference standard used for the AUC measure is a consensus standard, which is inherently not fully accurate. A limitation is that all three observers in the consensus group are from the same center; however, they did not receive their training in the same center. The ideal reference standard would be surgical confirmation of the presence of adhesions, but this is difficult to obtain for this study for two reasons. First, patients were included retrospectively, and not all patients underwent surgery. Patients with no evidence for adhesions evidently did not undergo surgery. Patients with a high complication risk, for instance, due to severe adhesions on cine-MRI or when considered unfit for surgery, did also not undergo surgery. Second, it is difficult to precisely relate the location of adhesions recorded during surgery to the location of adhesions on cine-MRI because of the different postures of the patient on the operating table during surgery. Moreover, the location of adhesions between organs can easily change relative to their projection on the abdominal wall during surgical manipulation. Thus, while the surgical ground truth can be considered the gold standard on patient-level, it can be problematic to relate surgical results to specific slices.

The analysis in this study was performed on slice-level. Clinically, this is meaningful, since it is more indicative of where adhesions are located than a patient-level analysis. If observers have high agreement on slice-level, they agree to a high degree on the location of adhesions, as well. However, Figure 5 shows (especially the right column) that even if observers agree on slice-level, there can still be disagreement about location. Figure 5 simultaneously highlights the varying annotation style, with some observers placing small boxes and others large boxes. Future studies could take this localization into account explicitly in the study design by carefully choosing the annotation type and process.

The scans for this study were selected in such a way that they represent a realistic cohort of scans that a radiologist would encounter in clinical practice. In our cohort, this means that approximately 30% of patients have no adhesions. In clinical practice, patients with a negative cine-MRI evidently do not receive a second surgery for their pain symptoms. Therefore, relying on surgical data as a reference standard would strongly skew the patient cohort toward patients with adhesions. This is another reason why this study intentionally opts for a consensus standard and also includes variability results that do not rely on a reference standard. Previously discussed work on the diagnostic accuracy of cine-MRI for adhesion detection suffers from the same problem. The entire cohort of Lang et al. underwent surgery to remove adhesions, giving indications for a high prevalence setting [7]. Indeed, only 4 out of these 89 patients (4%) had no surgical evidence of adhesions. Of these four patients, only one was classified correctly as negative by the observers, resulting in a specificity of 25%. Our study more faithfully emulates clinical practice by including 30% negative patients. The study by Zinther et al. includes 30 patients without surgical history and 30 with surgical history [16]. Interestingly, this study reports a sensitivity of only 24%, indicating that detecting adhesions might be harder when many patients without adhesions are included. 

A final limitation is the low number of cases included in the study. While our study indicates that general experience and cine-MRI experience improve performance, the results are not significant. On top of that, adhesion-specific experience surprisingly did not show significant effects in this study. This may be due to the low number of cases included or the low number of highly experienced observers in the study (especially the case for adhesion experience). The low number of cases was intentional, such that the time investment for the study was low. This allowed a larger team of international observers to join. A future study could benefit from more cases with perhaps a more carefully composed group of observers, including more observers with high specific experience. 

### 4.3. Future Perspective

Cine-MRI holds promise as an effective noninvasive modality for detecting abdominal adhesions. This study shows that observers can reach diagnostic accuracies of 0.75 after only a handful of training cases. Additionally, general experience and experience with cine-MRI improve agreement. Inter-observer agreement, however, is fair at best. AUC scores over the board are acceptable but leave room for improvement. This may be achieved by developing reporting guidelines for adhesion detection on cine-MRI, which are currently not available in the literature. More dedicated tutoring and training efforts may also help, creating a larger pool of experienced observers. Another route that can lead to improved agreement is the development of computer-aided diagnostics. Recent work shows that an inexperienced observer can match expert performance using a shearogram. The shearogram shows the shear along the abdominal wall, helping an observer by indicating sites with low shear as possible adhesions [17]. Further development of this approach is still necessary for adoption in clinical practice, as this method is limited to abdominal wall adhesions and requires elaborate manual segmentation. Recent work also shows promising results using deep neural networks to detect adhesions, lifting the abdominal wall restriction of previous methods [18]. 

## 5. Conclusions

This study indicates that cine-MRI is an effective modality to diagnose adhesions, with respect to a radiologist consensus panel. General and cine-MRI experiences improve reading cine-MRI, and observers without specific experience can reach acceptable diagnostic performance after a short tutorial. Observer agreement is fair at best, and AUC scores could be improved. Consistently interpreting cine-MRI for adhesion detection needs further research, for instance, by investigating reporting guidelines or artificial intelligence-based methods. 

## Figures and Tables

**Table 1:** Experience levels for all observers in the study. Observers marked with an asterisk (*) are in the consensus group. For other observers, bold entries indicate that they are in the high group for that experience category. For example, observer 15 is in the low-year, low-cine, and high-adhesion groups. The final column shows the clinical significance for each observer. For example, observers 6 and 8 consider adhesions clinically significant if they are at least 40% certain.

Observer	Years Experience	Cine Cases Reviewed	Adhesion Cases Reviewed	Clinical Significance Threshold
Observer 1 *	4	30	30	40%
Observer 2 *	30	100	100	80%
Observer 3 *	0	0	0	60%
Observer 4	**6**	**30**	**30**	40%
Observer 5	**15**	**100**	0	20%
Observer 6	**5**	**150**	0	40%
Observer 7	**7**	**50**	**50**	80%
Observer 8	**6**	**30**	**30**	40%
Observer 9	**17**	0	0	0%
Observer 10	2	15	**15**	40%
Observer 11	2	0	0	0%
Observer 12	2	0	0	60%
Observer 13	3	0	0	60%
Observer 14	3	**50**	0	40%
Observer 15	4	20	**20**	60%

**Table 2:** Inter-observer Fleiss’ κ, percentage agreement and the diagonally averaged AUC for all groups (low/high year: less/more than 5 years general experience, low/high cine: less/more than 30 cine-MRI studied, low/high adhesion: less/more than 1 adhesion cine-MRI studied). Values in brackets indicate the 95% confidence interval.

Group	Fleiss’ κ	% Agreement	AUC
consensus	0.28 [0.10, 0.44]	79.23 [74.32, 84.15]	-
low-year	0.06 [−0.01, 0.13]	56.28 [53.77, 58.91]	0.66 [0.57, 0.74]
high-year	0.34 [0.24, 0.42]	67.10 [64.59, 69.62]	0.72 [0.65, 0.79]
low-cine	0.04 [−0.03, 0.11]	56.28 [53.77, 58.91]	0.66 [0.58, 0.74]
high-cine	0.34 [0.24, 0.43]	67.54 [65.03, 69.95]	0.72 [0.65, 0.79]
low-adhesion	0.15 [0.09, 0.21]	57.53 [55.35, 59.72]	0.72 [0.66, 0.78]
high-adhesion	0.11 [0.02, 0.18]	56.39 [53.11, 59.51]	0.66 [0.57, 0.74]

**Table 3:** Cohen’s κ and percentage agreement values for all observers that completed the study twice. Observers marked with * are in the consensus group. Values in brackets indicate the 95% confidence interval. The observer numbers correspond to the numbers in Table 1.

Observer	Cohen’s κ	% Agreement
Observer 2 *	0.44 [0.22, 0.64]	78.69 [68.85, 86.89]
Observer 3 *	0.37 [0.20, 0.55]	75.41 [65.57, 83.61]
Observer 7	0.50 [0.29, 0.69]	78.69 [70.49, 86.89]
Observer 4	0.53 [0.36, 0.68]	75.41 [65.57, 83.61]
Observer 15	−0.12 [−0.18, −0.06]	75.41 [65.57, 83.61]

## Data Availability

The data presented in this study are available on request from the corresponding author. The data are not publicly available due to the privacy of sensitive information contained in the data.

## Supplementary Materials

The following supporting information can be downloaded at: https://www.mdpi.com/article/10.3390/jimaging9030055/s1, Figure S1: Observer tutorial; Figure S2: ROC operating points; Videos S1–S3: Annotated examples.
