# Supplementary material for: Inter- and Intra-Observer Variability and the Effect of Experience in Cine-MRI for Adhesion Detection
Source: J Imaging. 2023 Feb 23;9(3):55. doi: 10.3390/jimaging9030055 (PMC10054690; doi:10.3390/jimaging9030055)

Observer tutorial

All observers had access to an online tutorial study of 4 cases, with a short text explaining the most important aspects of adhesion detection. The study is openly available here:

https://grand-challenge.org/reader-studies/adhesion-cine-mri-tutorial/

Online observer environment

Below, a screenshot of the online environment that was used to run this observer study.

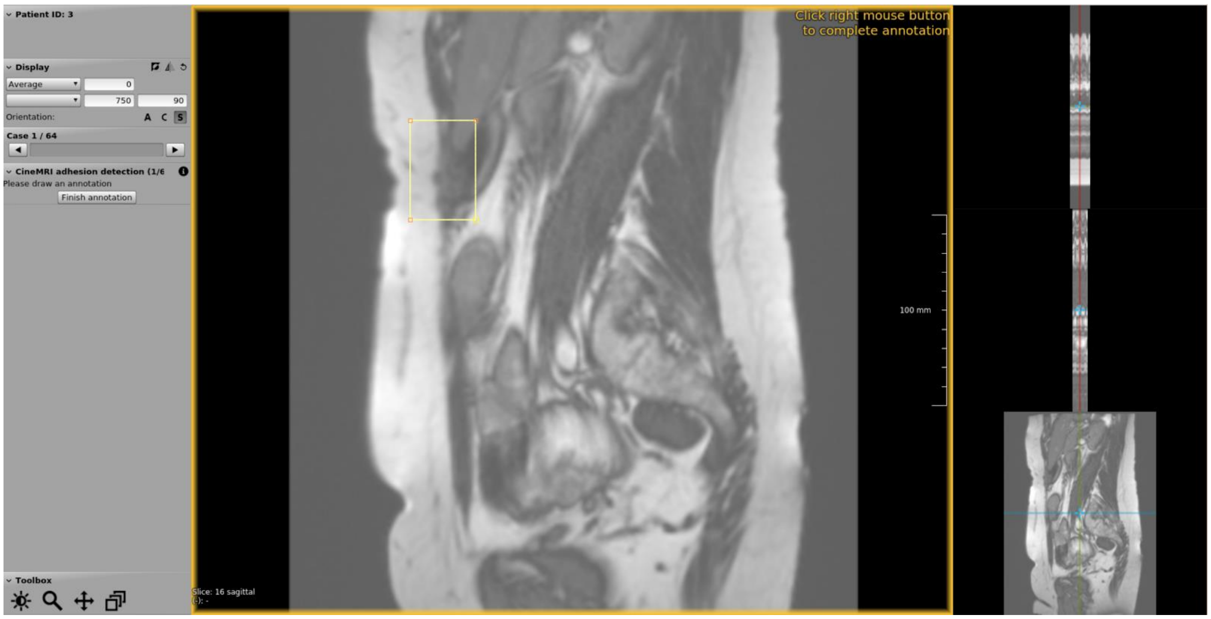

Supplement: Supplementary file 1 [file jimaging-09-00055-s001.zip › Figure S1.pdf]
