# Supplementary material for: Inter- and Intra-Observer Variability and the Effect of Experience in Cine-MRI for Adhesion Detection
Source: J Imaging. 2023 Feb 23;9(3):55. doi: 10.3390/jimaging9030055 (PMC10054690; doi:10.3390/jimaging9030055)
Supplement: Supplementary file 1 [file jimaging-09-00055-s001.zip › Figure S2.pdf]

Figure S2: ROC operating points

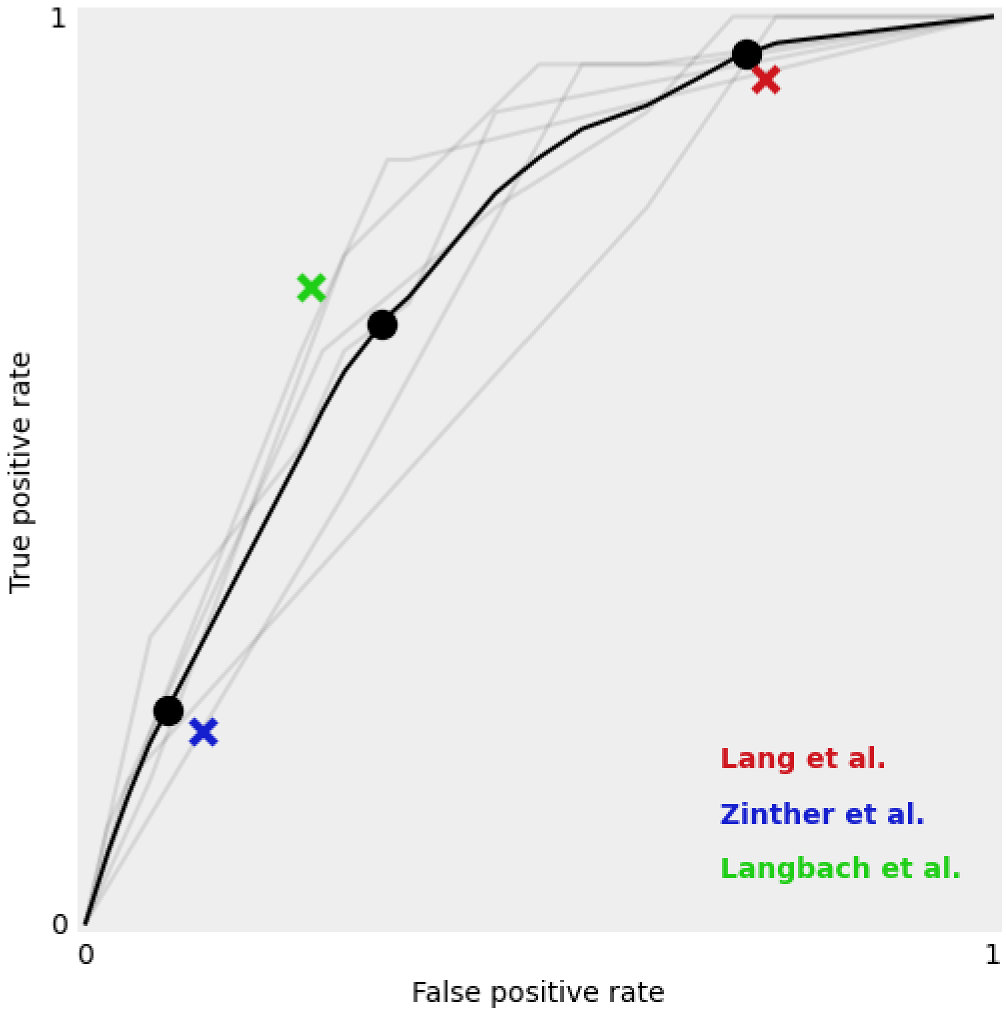

The average AUC for the high-year group, with three operating points (black dots) matching results in literature mentioned in the Discussion (crosses).
